# Supplementary material for: Are carbon emissions trading and green financial instruments synergistic? -Comprehensive quantitative research based on content analysis
Source: PLoS One. 2024 Mar 7;19(3):e0298601. doi: 10.1371/journal.pone.0298601 (PMC10919601; doi:10.1371/journal.pone.0298601)
Supplement: S1 Data — (ZIP) [file pone.0298601.s001.zip › Data/地级市Z_F工作报告.zip!/╣π╢1⁄2/╜¡├┼╩╨/└┤╫╘╣1⁄2╓┌║┼í╛┬φ┐╦╩2╛▌═°í┐_2003.docx]

各位代表：

我代表市人民政府向大会作政府工作报告，请予审议，并请政协委员和其他列席人员提出意见。

过去五年工作回顾

　　本届政府任期的五年，是战胜困难加速前进的五年。五年来，在省委、省政府和市委的领导下，在市人大及其常委会和市政协的监督支持下，我们高举邓小平理论伟大旗帜，认真贯彻“三个代表”重要思想和党的十五大、十六大精神，以“增创新优势，更上一层楼，率先基本实现社会主义现代化”为总目标总任务统揽工作全局，紧紧依靠全市人民，克服亚洲金融危机冲击、世界经济增长放慢等因素带来的困难，坚持以经济建设为中心不动摇，大力推进物质文明、精神文明和民主法制建设，较好地完成了市第十一届人民代表大会确定的各项任务。

　　一、经济综合实力显著增强

　　２００２年全市国内生产总值为６６０．８２亿元，五年年均增长１０．３％，人均国内生产总值达２０９０美元。地方财政一般预算收入２５．７４亿元，年均增长１２．３８％。三大产业结构比例由１３：４９：３８演进为１１：４８：４１。

　　——工业结构不断优化。大力推进科技创新和体制创新，涌现出一批有较强实力的支柱产业和优势企业。五年全市工业总产值增长８２．０１％，去年达到１４２５．９６亿元。摩托车整车年产量由２４．３８万辆增加到８７．５４万辆，居全省第一位；纺织化纤服装业规模扩大，开平市被授予“中国纺织产业基地市”称号；空调器年产量从１８．３万台增至６９．３万台；造纸及纸制品业引进了新加坡金鹰造纸项目，维达纸业居全国同行业前列，我市成为华南地区最大的造纸基地已具雏型。抓质量、创名牌工作取得进展。“维达”、“豪爵”、“金羚”等被评为中国驰名商标或中国名牌产品，３２个产品和１６个商标分别被评为省级名牌产品和著名商标。全市获国际质量认证的工业企业由５０家增加到１５０家。以企业为主体的技术创新机制进一步完善，我市被列入国家技术创新试点城市和国家信息化试点城市。传统产业得到改造提升，五年工业企业技改总投资１７８．４７亿元。全市高新技术企业从４３家发展到８４家，民营科技企业从４１家发展到１６０家，形成了以电子信息、光机电一体化、生物工程、精细化工、新能源和新型材料为重点的高新技术产业，高新技术产品产值占全市工业总产值的比重由五年前的１２．８％提高到１５．６％。工业经济效益综合指数为１１７．９５％，比１９９７年提高了３１．３５个百分点。信息化综合指数为５０．５３％。

　　——农业产业化水平提高。全市粮经作物种植比例由１９９７年的７０：３０调整为５０：５０，发展农业龙头企业１２４家，农业现代化示范基地建设取得新进展，辐射带动能力进一步增强。全市主要农作物良种和畜禽良种覆盖率达到９３％以上；建立了３８个无公害农产品生产基地，８个农产品获得国家绿色食品认证。实施强镇富民战略，发展“一镇一品”的区域特色经济，蓬江区的摩托车、新会区司前镇的不锈钢制品、开平市水口镇的水暖卫浴器材等被列为省级专业镇区技术创新试点。全市乡镇企业总产值由１９９７年的６５１．８亿元增至１１０６．３亿元。镇级财政收入１０．８８亿元，占全市财政收入的比重上升到４２％，有３２个镇财政收入超千万元。

　　——第三产业发展加快。以发展休闲度假旅游为重点，全市初步形成了温泉、沙滩、碉楼和侨乡人文景观四大旅游品牌，２０００年获“中国优秀旅游城市”称号，开平立园、新会圭峰山风景名胜区、恩平金山温泉度假村被评为国家４Ａ级景点。引进外资建成五邑蒲葵高尔夫球场；成功举办了两届中国（江门）侨乡旅游节。旅游总收入年均增长１０．６％；去年旅游外汇收入突破１亿美元。大力发展现代流通服务业，引进了人人乐、新一佳等著名超市和大型商场，提升了商贸流通业的竞争力和整体素质。培育和发展了汽车、摩托车、农副产品等一批专业批发市场。全市社会商品零售总额年均增长１１．５％。房地产业发展水平提高，五年新建住宅面积１１５３．９３万平方米，房地产投资占三产的４０．６３％。信息建设进展快速，通信设施全面实现程控化、光纤化和数字化，固定电话普及率从１９９７年的每百人１２部增至２７部，移动电话用户由１０．２６万户增加到１３６．９１万户，互联网用户３３．７３万户。同时，金融保险业、中介服务业、运输仓储业、社区服务业和文化教育产业也有较大发展。

　　——民营经济成为新的经济增长点。制定和组织实施鼓励民营经济发展的政策措施，进一步放开民资投资领域，积极改善各项服务，营造良好的投资发展环境。去年全市民资投资３２亿元个体工商户总数突破１０万户，私营企业总数超过１００００户，分别居全省第四位和第六位。民营经济在国民经济中所占比重由１９９７年的４６％上升到去年的５３．６３％。全市私营企业户均注册资金由１９９７年的４８．３５万元提高到２００２年的６７．５６万元，年产值超亿元的私营企业增加到１０家，缴税５００万元以上的１５家，新会天健集团和鹤山建昌集团进入全国５００强私营企业行列。

　　二、对外开放上新水平

　　我们坚持把招商引资作为经济工作的重中之重来抓，设立市政府行政总汇、“马上办”和“投诉中心”，改革审批制度，简政提速，开通市政府网上投资业务报批系统。不断创新招商方式，先后举办了经贸洽谈暨摩托车工业博览会、经济技术贸易洽谈暨机电产品博览会等重大招商活动；与世界银行合办了投资环境推介会；组织小分队到美国、德国、韩国、日本等国以及台湾等地招商，开展网上招商、专业招商和委托代理招商，引进了一大批项目。开平市中晖复合纤维母粒、蓬江区大长江、新会区中集、现代等４家企业进入全国外商投资企业５００强。发展工业园区经济，全市工业园区五年共引入投资项目１３７０多个，外资投入１６．３亿美元，民资投入９５．３亿元。这五年，全市累计批准利用外资项目７４００多个，兴办三资企业１４００多家，实际利用外资３６．２７亿美元，相当于１９７９－１９９７年１９年的总和。

　　对外贸易不断扩大。五年来全市累计出口总额达１３４．７５亿美元，比上五年增长２４．２％，出口超千万美元的企业７１家。出口经营主体呈现多元化，民营进出口企业从空白发展到１４５家。优化出口产品结构，机电产品年出口比重由１９９７年的３３．８％提高到５２．９５％。出口市场多元化取得突破，远洋贸易比１９９７年增长了４６．３４％，占出口比重提高到５３．５８％。

　　三、各项改革向纵深推进

　　以国企改革为重点的各项改革向纵深推进。大力吸引外资民资对国企进行投资性重组，全市１９０４户国有企业完成改制面逾８０％。国有大中型企业基本建立现代企业制度，实现了三年扭亏脱困两大目标。以养老、失业、医疗保险为重点的社会保障体系覆盖面进一步扩大，参保率达９６％以上，离退休人员养老金实现１００％社会化发放。

　　政府职能进一步转变。推进政企、政资、政事分开，继续深化市、县、镇三级党政机关机构改革；清理、修订和废除不符合世贸组织规则的规范性文件；改革审批制度，把原有１１６６项审批和核准事项，削减至２１１项，减幅达８２％。在干部人事制度中引入和建立、完善竞争激励机制。组织开展“三服务一满意”活动，促进廉洁、高效、勤政、务实良好政风的形成。建立公共财政框架，实行政府统一采购和国库集中支付制度，全面推进预算外资金“收支两条线”，调整了市区财政管理体制。防范化解地方金融风险工作积极稳健，财政金融形势比较稳定。

　　此外，全面完成农村土地延长承包期工作，撤并乡镇１９个，推进农村税费改革，共取消２７２项收费，降低收费６７项，减轻企业和群众负担４．７８亿元。整顿市场经济秩序，加强了市场管理。科技、教育、文化、药品等管理体制改革都有新突破。

　　四、城市建设成绩显著

　　基础设施建设取得重大进展。五年来，全市累计固定资产投资５４７．９５亿元，是我市历史上固定资产投资最多的时期。新建高速公路１７４．５公里，公路密度从１９９７年的每百平方公里５５．９６公里提高到６６．０３公里，佛开、江鹤、新台、西部沿海等高速公路建成通车，开阳高速公路即将建成，江中、江珠高速公路已经或即将动工。新会港被定为国家一类口岸，银洲湖成为对外轮开放水域。台山鱼塘港工程建设全面铺开。台山电厂首期工程１号机组今年将建成发电。按期超额完成省人大“关于小型水库除险加固”议案任务，加固江新联围、锦江水库等一批水利设施。

　　大力推进城市和镇村规划建设。江门中心城市完成行政区划调整，总面积扩大为１８１８平方公里，人口１３０万人，为整合区域资源、实现区域协调发展创造了条件。以江门市区为中心，四个县级城市为骨干、７３个建制镇为节点的城镇体系初步形成，全市城市建成区面积从１８７．５６平方公里发展到２３０．３５平方公里，全市城市道路１６４２公里，人均公共绿地面积９．４４平方米，绿化覆盖率从３４．０９％提高到３７．６８％。２００２年江门市荣获“国家园林城市”称号。

　　五、人民生活进一步改善

　　城乡居民生活从小康向基本富裕迈进。２００２年全市在岗职工人均年工资收入１１１７０元，农民人均年纯收入４７９３元，分别比１９９７年增长了３７．３％和２４．５％。去年末全市城乡居民储蓄存款余额５７８．３５亿元。人均居住面积五年来增加了４．２平方米，达１８．２３平方米。摄像机、电脑、汽车等高档消费品陆续进入百姓家庭。城镇人均年交通通信娱乐教育文化服务消费占总消费比重从１９９８年的２１．５％提高到３４％。

　　加强社会就业和下岗职工再就业工作。五年累计安排就业１６．７万人；城镇登记失业率控制在省要求的指标以内。城乡居民最低生活保障制度逐步健全，保障水平不断提高。１９９７年实施城市最低生活保障制度，１９９９年扩大到农村，城乡低保救济分别为人月均２６０元和１３０元。加强扶贫工作，开展农村挂钩扶贫和对城镇困难群众送温暖活动，切实解决“四难”问题，社会弱势群体获得基本生活保障。

　　六、实施可持续发展战略取得实效

　　依法保护和合理开发利用水资源以及土地、森林、矿产、海洋等重要资源。加大环境综合治理力度，实现甘化厂制浆红液治理达标排放，新建了文昌沙污水处理厂和鹤山杰洲污水处理厂，城市污水处理率从２．７３％提高到２８．４９％。加大工业污染治理力度，全市１５３０家工业企业实现基本达标排放。小型饮食店全面使用清洁能源。加强跨市河流水质保护工作，２４３万亩生态公益林得到有效保护。全市环境考核综合指标由６４．１４分提高到８３．９６分，进入全省优秀行列。

　　人口计划生育工作逐步纳入了法制化、规范化、制度化的轨道。 ２００２年全市人口出生率为８．９８‰，比１９９７年下降５．９９个千分点；人口自然增长率为３．５１‰，下降了６．１１个千分点；计划生育率达到９０．０２％，提高了７．７６个百分点。

　　七、精神文明建设和社会各项事业有新进展

　　全面加强社会主义精神文明建设。我们坚持“两手抓、两手都要硬”的方针，以创建文明单位、文明社区、文明村、文明城市为载体的精神文明创建活动取得明显成效，开平市被评为省文明城市，江门、新会被评为省“创建文明城市先进单位”。江门连续两次荣获“全国双拥模范城”称号。

　　教育事业长足发展。全市完成２７５所薄弱学校的改造，建成省和地市一级中小学总数比例居全省前列。巩固“普九”成果，推进“普高”工作，全市初中毕业生升学率由１９９７年的６７．７５％上升到２００２年的８３．４３％每万人口有高中阶段在校学生２９５人。职业教育继续名列全省前茅，建成国家级和省级重点职业中学各８所，中等职业学校学生一次性就业率达９０％以上。学前教育、成人教育、特殊教育也有新发展。五邑大学办学水平在省内地方大专院校中继续保持先进，并不断发展提高。全市适龄青年高等教育入学率达２１．３９％。制定和落实引进高层次人才政策，全市每十万人口大专以上人数由１４７４人增加到２４５８人。

　　文化建设创佳绩。发展具有地方特色的侨乡文化、民俗文化、旅游文化、广场文化和社区文化，大力活跃群众文化，新会、台山、开平被评为“中国曲艺之乡”；创作了一批群众喜闻乐见的优秀作品，五年来全市有３２６件作品获得国家和省文化部门的奖励；广播电视实现全市光纤联网；开展华侨博物馆资料征集工作，新闻、出版、地方志、档案等各项工作取得新成绩。

　　卫生事业取得新进步。深化医疗卫生体制改革，建立药品集中采购招标制度。开展社区卫生服务，推进社会无偿献血。新建了一批医疗卫生基础设施，改善了群众就医条件。预防保健工作全面加强，１９９８年提前两年实现了农村初级卫生保健规划目标，２００２年设立市疾病控制中心和市卫生监督所，依法加强公共卫生监督执法工作。广泛深入开展全民爱国卫生运动，最近几个月全力以赴抗击非典型肺炎，有效控制了疫情。

　　体育运动水平有新提高。各类群众性体育活动蓬勃开展，全市成年人国民体质检测合格率超过全省平均水平。五邑各市都被评为全国体育先进县（市）。成功承办了世界杯花剑赛、亚洲台球锦标赛、世界女子围棋锦标赛、陈杰恒杯全国游泳锦标赛等一系列重大体育赛事。竞技体育水平不断提高，在第十一届省运会中，江门代表团共夺奖牌１３３．５枚，其中金牌５２枚，比上届翻了一番。江门输送的优秀运动员在全国和国际体育比赛中取得喜人成绩，陈小敏夺得奥运会举重金牌。

　　社区建设全面启动。完成了以社区调整划分和社区居委会成员民主选举为主要内容的城市基层管理体制改革，社区民主自治体制基本形成，社区服务逐步完善。全市现已建成社区服务中心６个，社区服务站点１４９个，便民服务点５８２个，基本实现了城市管理重心下移的转变。

　　认真落实归侨侨眷保护法，加强与海外华侨社团的沟通联系，积极发展侨务事业。民政、宗教、人防、残联、老龄、气象等各项事业都有新发展。

　　八、民主法制建设得到加强

　　深入开展普法教育，落实依法治市五年规划。推进依法行政，加强行政执法队伍建设和行政执法监督，建立健全执法过错责任追究制度和行政管理公示制度。各级政府自觉接受同级人大的监督，切实办好人大议案、人大代表建议、政协委员提案；注意经常听取工青妇、民主党派、无党派人士、工商联和人民团体的意见，推进重大决策的民主化、法制化。认真做好人民来信来访工作。积极推行政务公开，开展“万人评机关”活动。完成撤销农村管理区建立村民委员会的工作，理顺基层管理体制。

　　加强社会治安综合治理。坚决打击“黄、赌、毒”等违法犯罪活动，开展专项整治和严打斗争，坚决取缔“法轮功”邪教组织，大力整治非法传销活动，及时疏导和处置各类纠纷，维护社会稳定。抓好安全生产，对重点行业的防火、安全工作开展经常性检查，加强交通安全宣传和秩序管理，有效消除安全事故隐患。

　　加强政府廉政建设。建立健全廉政建设和反腐败斗争的领导层级责任制，突出抓好领导干部的廉洁自律、查处大案要案和纠正行业不正之风，政府机关作风明显好转。

　　回顾本届政府五年来的工作，我们深深地体会到：必须始终坚持以邓小平理论和“三个代表”重要思想为指导，把上级指示精神与本地实际结合起来，不断探索创新我市发展思路；必须坚持以经济建设为中心，解放思想，与时俱进，创造性地开展工作，用发展的办法解决前进中的困难和问题；必须坚定不移地推进对内对外开放，充分利用国际国内两个市场、两种资源加快发展；必须以制度创新和科技创新为动力，大力推进以国企改革为核心的各项改革，大力发展民营经济，以改革促发展，以发展保稳定；必须始终坚持物质文明与精神文明、政治文明建设一起抓，法治与德治相结合，推进社会的全面进步；必须始终把改善人民群众生活作为发展的立足点，努力增加城乡居民收入，关心和解决困难群众的生产生活，调动海内外一切积极因素，加快侨乡现代化建设。

　　各位代表：本届政府五年来所取得的成绩，是邓小平理论和“三个代表”重要思想指引的结果，是省委省政府和市委正确领导的结果，是各级人大和政协监督支持的结果，是全市人民和中央、省驻江各单位及各驻军部队共同努力的结果，是各民主党派、社会各界人士以及广大港澳台同胞、海外侨胞关心支持的结果。在此，我代表本届政府，向关心支持江门经济社会发展的同志们、朋友们、港澳台同胞和海外乡亲表示崇高的敬意和衷心的感谢！

　　我们清醒地看到，在我市经济社会发展中，还存在许多有待解决的问题，主要是：经济增长的速度与效益还不够高，产业的总体竞争力还不够强；财政收入占ＧＤＰ的比重偏低，历史原因形成的各级政府的债务负担还比较重；金融风险遗留隐患未完全消除；国企改革攻坚力度还不够大，体制性障碍仍然存在；社会就业压力加大；城市化进程仍需加快；机关作风建设和廉政建设存在薄弱环节。对这些问题，我们必须引起高度重视，采取有效措施，切实加以解决。

　　今后五年的奋斗目标和２００３年的工作建议

　　今后五年是我市全面建设富裕文明小康社会、基本实现社会主义现代化的关键时期，是二十一世纪头二十年的战略机遇期。经济全球化和科技经济一体化趋势在增强，国际间新一轮产业转移在加快，粤港澳经济合作在加深；我国加入世贸组织后，政治稳定，经济持续增长，继续实行扩大内需的方针政策，投资吸引力为世界之最。而我市随着交通条件的进一步改善，江中、江珠、开阳、江肇高速公路、滨江大道和广珠铁路将陆续开通，作为珠三角西岸中心城市的区位优势将有更大的提升。新的历史性的发展机遇展现在我们面前。我们必须抢抓机遇，奋力开拓，聚精会神搞建设，一心一意谋发展，努力开创现代化建设的新局面。

　　今后五年政府工作总的指导思想是：以邓小平理论、“三个代表”重要思想、党的十六大精神和胡锦涛同志视察广东讲话精神为指导，以全面建设富裕文明小康社会、基本实现社会主义现代化为目标统揽全局，狠抓发展第一要务，按照市委提出的工业强市、民资外资富市、科教兴市、环境优市的发展战略，加快推进工业化、信息化、城镇化和农业产业化，增强综合经济实力和可持续发展能力；不断改善人民生活，营造优良的人居和创业环境，建设具有侨乡特色的人与自然和谐统一的山水园林生态城市；推进社会主义政治文明和精神文明建设，全面建设富民强市新侨乡。

　　今后五年我市的主要目标和总体要求是：全市国内生产总值突破１０００亿元，年均增长１１％以上；全社会固定资产投资总额超１０００亿元；地方财政一般预算收入增长速度高于国内生产总值增长的速度。产业结构进一步优化，高新技术产品产值占工业总产值的比重提高到３０％以上，信息化综合指数达到６０％。城乡一体化进程加快，城镇化水平达到６０％。全面完成国有企业改革任务，非公有制经济在国民经济中的比重提高到９０％以上。城乡居民可支配收入不断提高，城镇居民人均收入和农民人均纯收入年均分别增长８％和５％，贫困地区群众生产生活条件明显改善。创建“国家卫生城市”、“国家环保模范城市”、全省文明城市。努力实现经济社会的协调发展，即速度与结构、质量、效益的协调发展，外源型经济与内源型经济的协调发展，区域经济的协调发展，经济发展与人口、资源、环境协调发展，物质文明、政治文明、精神文明协调发展。

　　２００３年是我市贯彻党的十六大精神，实现“十五”计划的重要一年。今年我市经济社会发展的预期目标是：国内生产总值增长１１％，地方财政一般预算收入增长１２％，社会消费品零售总额增长１２％，固定资产投资增长１３％，外商直接投资增长１５％，外贸出口增长８％，居民消费价格持平或略有上升，城镇登记失业率控制在３．５％左右，人口自然增长率控制在５．７‰以下。

　　为实现上述目标，建议新一届政府着力抓好以下工作：

　　一、 推进经济结构调整，提高经济增长质量

　　要坚持速度、结构与质量、效益相统一，努力实现经济增长从量的扩张到质的提高。

　　不断优化工业结构，走新型工业化道路。加快发展机电产品、造纸及纸制品、纺织服装、食品、电子信息五大制造业基地，打造摩托车、纸业、食品、家电、机械装备、电子产品和金属制品等名牌产品，做大做强工业龙头企业。加大以信息技术改造传统产业的力度，推动企业普及计算机辅助设计技术和信息管理技术，着力扶持电子信息、生物制药、新材料、光机电一体化等重点项目，加强区域经济技术创新体系建设，搞好对企业的科技服务，增强地区、行业和企业的核心竞争力。大力培育发展民营科技企业，鼓励发明专利，加强科技对外合作与交流。加大企业技改项目和新产品开发项目的投入，努力提升产业素质。

　　扩大产业“族群”，促进区域协调发展。大力推进沿海、沿湖、沿江、沿路组团式工业园区的开发建设，重点加快市高新技术产业开发区、新会银洲湖、江沙工业走廊、西部沿海高速公路沿线、３２５国道沿线等区域开发，以及市、区、镇重点工业园区的开发建设。积极引导发展区域特色经济，促进蓬江区的摩托车及零部件、新会区司前镇的不锈钢制品和大敖镇的集装箱、开平市水口镇的水暖卫浴设备、恩平市的麦克风及电声器材、鹤山市的制鞋等特色经济做大做强，形成产业链条，建设在国内外具有重要位置的制造业名市、名镇。

　　深化农业结构调整，努力增加农民收入。我市农业和农村经济发展要紧紧围绕增加农民收入这个中心，大力推进农村经济结构调整，着力在农业产业化、农村工业化、城镇化和农村劳动力转移上下功夫。要推进农业生产的品牌化、标准化、规模化，因地制宜地发展一批具有市场潜力的名、优、特农产品和安全食品，重点建设十大无公害基地和绿色食品基地。继续优化种植结构，做大做强畜牧业、林业、水产业和海洋渔业，发展园艺业，扶持旅游农业。积极开发、引进和推广农业新品种。扶持发展农产品加工业，提高农产品附加值。大力发展农业龙头企业，搞好现代农业示范基地建设，壮大科技示范村、示范户、示范基地，提高辐射带动能力，推进农业产业化经营。办好农业信息网，加强农副产品市场建设，扩大农产品的市场流通。调整市、县两级农科机构，全面启动农业科技创新“七大工程”。拓宽农业投入渠道，搞好农业招商引资，鼓励外资民资兴办农业项目。继续推进扶贫开发。贯彻“多予、少取、放活”的方针，减轻农民负担，增加农民收入。

　　以旅游业和现代物流业为重点，加快发展第三产业。旅游业要进一步开发优质温泉、滨海沙滩、侨乡风情、森林生态等旅游资源，努力培育休闲度假品牌，打造旅游精品，创更多的国家４Ａ级旅游景点，办好在开平举办的第三届中国（江门）侨乡旅游节。大力发展现代流通业，特别是连锁经营、大型超市、现代物流业、中高档批发市场和现代会展业；继续发展房地产业，抓好规划和市场调控，提高物业管理水平，促进房地产稳步健康发展。开展会计、审计、法律咨询、建设监理、房地产交易、职介、信息咨询等社会中介服务，积极发展金融保险、仓储运输、智力开发、体育健身、卫生保健、文化娱乐和社区服务业，拓宽第三产业发展的新领域。鼓励信贷消费，改善消费环境，引导居民消费预期，开发农村消费市场，使其形成新的消费热点。

　　二、 营造优良环境，加快发展外源性经济和内源性经济

　　充分发挥江门的侨乡、区位、资源、产业优势，实现外源经济和内源经济的协调发展。

　　发挥“侨”的优势，着力发展外资经济。华侨众多是我市发展外源性经济的一大优势。抓好以侨引资，把江门侨乡优势转化为投资发展优势，过去是今后仍然是我市加快发展的重要法宝。要创新招商模式和招商策略，积极推行代理招商、网上招商、小分队招商等，重点瞄准国内外的大企业、大财团，提高招商引资的实效。侨务外事部门要密切配合，做好引资引智工作。当前重点抓好金鹰造纸、华尔润玻璃、大冶摩托、台山泰立斯国际电子、鹤山捷士克汽车配件、“李锦记”食品、新会罗坑冠华、中集集装箱、吉事多卫浴、雅图士扩产等大型项目的上马和增资扩产。

　　发挥民营经济的优势，着力培植新的经济增长点。要把民营经济作为加快我市经济发展的潜力所在和希望所在，大力培育和发展。坚持“政治平等、政策公正、法律保障、放手发展”的方针，不限发展比例、不限发展速度、不限经营方式、不限经营规模，激活本地民资，吸引外地民资。重点扶持民资出口创汇企业、科技企业、农产品深加工企业以及扩大就业型和规模经营型企业，鼓励民营企业投资基础设施建设、城市公用事业以及教育、卫生、文化、体育等领域，鼓励民营企业以多种形式参与国企的重组改造，努力为民营企业家营造“社会上有地位、政治上有荣誉、经济上有实利、管理上有秩序”的良好发展氛围，切实保护私人投资的合法权益。进一步做好服务，发挥中小企业信用担保中心的作用，解决民营企业融资难的问题。

　　发挥机电等支柱产业的聚集优势，着力开拓国内外市场。发挥我市机电、纸业等品牌的优势，充分运用各种推介方式，进一步开拓和抢占国内外市场。要搞好重点产业园区的规划、建设和招商工作，加快进园项目的基建和投产达效，使之成为拉动我市工业经济的重要增长点。

　　发挥现有外贸出口优势，着力开创外贸出口新局面。要根据入世后的新形势，努力优化出口产品结构和市场结构，扩大一般贸易出口，增加远洋贸易。实施市场多元化战略，在巩固港澳、欧美日、东南亚等传统市场的同时，积极开拓俄罗斯、中东、非洲、拉美等新市场。鼓励和支持有条件的企业“走出去”，发展对外加工业务，增强产品出口竞争力，扩大对外承包工程和劳务合作业务，带动成套设备和技术出口，积极发展服务贸易和国际旅游业。深化外贸体制改革，发展壮大技工贸一体化的外向型企业，全力支持更多的民营生产企业取得进出口经营权，使民营企业逐步成为外贸出口的主力军。

　　发挥江门地缘、人缘、资源等优势，着力改善投资环境，扩大招商引资成果。认真落实优惠政策，千方百计降低投资者的初始成本和比较成本。深化行政管理改革，减少审批事项和环节，健全首问责任制，实行机关作风行政首长负责制，努力创造省内乃至全国堪称一流的行政管理效能和办事效率。

　　三、深化改革，创新体制

　　在深化改革中要坚决打好四个攻坚战：

　　打好以产权为核心的国企改革攻坚战。国企改革要以产权改革为核心，鼓励民资外资参与国有企业改造重组，加快国有企业民营化步伐，形成侨乡经济以民资外资为主体的格局。今年要基本完成国企转制工作。要搞好改革配套，特别是做好企业改制后的人员安置补偿和债务处置工作，抓好下岗人员的再就业和生活保障，确保社会稳定和经济发展。加强产权交易市场建设，规范产权交易行为。加快上市公司国有股权重组，优化股权结构，打造企业发展新的增长点。

　　打好公有资产管理体制改革攻坚战。进一步改革公有资产管理体制，建立起权利、义务和责任相统一，管资产和管人、管事相结合的公有资产监督管理体制，完善公有资产保值增值考核办法，建立科学合理的经营者业绩考核和分配体系，提高国有资产运营效益。

　　打好财政管理体制改革的攻坚战。构建公共财政基本框架，加强依法征管税收，深化财政管理体制改革。调整财政支出结构，提高社会保障支出的比重，保工资、保教育、保农业、保稳定。推进部门预算改革，全面推行国库集中支付，扩大政府公开采购，加强预算外资金管理，完善收支两条线制度，确保财政增收和实现收支平衡。

　　打好整顿市场经济秩序的攻坚战。进一步规范市场经济秩序，继续开展以食品、药品、农资等关系群众生活和生产安全的商品为重点的专项整治，打击制售假冒伪劣商品和传销行为，保护消费者与经营者的合法权益。加强社会信用体系建设，提高政府公信力；建立企业信用评价、监督管理、责任追究等信用制度，促进企业守法经营。

　　此外，要继续深入推进城市管理、教育、科技、文化、医疗等管理体制改革，消除各种体制性障碍，促进经济社会发展。

　　四、加强城市环境建设，增创可持续发展优势

　　要按照市委提出的“三大组团”和“五大工程”的发展思路，加大城市建设力度，进一步增强中心城市和中心镇的辐射力。

　　承接广佛，加快基础设施建设。要从更好地融入珠三角经济圈，提升我市区位优势的高度，加快道路、港口、电力、通讯等基础设施建设。一是打造四通八达、快捷方便的交通网络，构筑现代化交通新格局。重点抓好开阳、江中、江珠、江肇、江鹤二期高速公路、滨江大道、珠三角轻轨江门段、广珠铁路江门段等项目的建设。二是加快新会港和台山鱼塘港建设，疏浚崖门出海航道和劳龙虎水道，增创沿海临港经济新优势。三是加大电力、供气、信息网络建设的力度，重点加快台山电厂、输变电工程、城镇液化气供应网、信息高速公路网建设。四是加强水利设施建设。重点抓好江新联围整治等项目，提高防险抗灾能力。

　　整合资源，加强中心城市的规划建设管理。要以建设现代化滨江临港大城市为目标，做好江门市城市总体规划的修编工作。江门市区以建设滨江新城（含北新区）为亮点，搞好老市区与新会区的功能布局和资源整合，扩大城市发展空间，优化城市功能结构。提高城市综合管理水平，加快“城中村”改造步伐，推进城市管理重心下移，形成市、区、街道（镇）三级相互衔接、合理分工、规范高效的城市综合管理体制。精心经营城市，全面整合城市土地、交通、能源、水务、房产等资源，在盘活有形资产的同时，积极探索把分散的处于非经营状态的国有资产物业集中统筹运营，鼓励外资民资投资建设城市公共设施，拓宽城市建设的筹资渠道。改革公用事业投资运营机制，最大限度地降低运作成本，提高资金效益，积极引入ＢＯＴ（建设－运营－移交）和ＴＯＴ（移交－运营－移交）等有效模式，推行公用事业特许经营权招标，加快城市建设。

　　发展县域中心城市，大力培育中心镇。各市要高标准、高起点搞好城区和中心镇的规划和建设，完善城市功能，形成以各市城区为重点、中心镇为骨干的城镇化网络体系。把县域中心城市、中心镇建设与专业市场、工业园区、基础设施建设有机地结合起来。开展土地整理和易地置换，拓宽城镇建设和二三产业空间。大力推进村镇建设，坚持以工业化带动城镇化，以产业聚集发展促进城镇发展。

　　加强环境保护工作，实现可持续发展。各级政府要切实落实环保目标责任制，确保环境质量达标。以保护饮用水质为重点，实施流域水质保护规划和区域水污染整治计划，强化跨市河流交界断面水质达标管理。全面治理河涌，限期解决污染问题。各市和中心镇要加快生活污水处理厂建设，提高城镇生活污水处理率。严格控制新上污染项目，严格监管危险废物和进口废物。推行清洁生产和循环经济，控制污染物排放总量，确保工业污染源全面达标。加强对生产生活噪音的监管。继续抓好生态示范区和生态示范村建设，巩固发展造林绿化成果，提高森林覆盖率，重视自然保护区、森林公园建设及野生动植物保护。坚持土地用途管制，严格控制非农建设用地，实现耕地占补平衡。保护海洋环境和海岸带、珊瑚礁等生态系统。建立矿山生态环境恢复治理示范区。通过环境整治，促进经济社会的可持续发展。

　　五、做好就业和社保工作，提高人民生活水平

　　进一步改善人民生活。在实现经济有效增长的同时，努力增加城镇职工和农村农民的可支配收入。进一步规范和完善公务员岗位津贴发放，建立正常的工资增长机制，逐步提高公务员和享受公务员待遇人员的收入。扩大中等收入者的比重，提高低收入者水平，促进共同富裕。大力发展公共交通、社区服务和教育文化产业，丰富城乡居民物质文化生活。

　　抓好社会就业工作。通过发展服务业、劳动密集型企业、社区服务业和非公有制经济，广开就业门路，拓宽就业渠道。引导择业者转变就业观念，鼓励通过非全日制、临时性、季节性、弹性工作等灵活多样的形式实现就业。开展多种形式的再就业培训，完善就业服务体系，为下岗失业人员提供职业介绍、就业指导、职业培训等“一站式”服务。

　　完善社会保障体系。以养老、失业、医疗保险为重点，建立和完善制度规范化、资金多元化、管理服务社会化的社会性保障体系。积极推行农村最低生活保障制度。依法扩大社会保险基金的征管面，强化征缴，规范支出，进一步健全社会保险基金监管体系，严格规范社会保险业务管理。

　　要关心群众疾苦，切实帮助城乡困难群众解决就业难、就学难、就医难、打官司难等突出问题。继续推进扶贫开发工作，积极发展城乡社会救济和社会福利事业，逐步提高重点优抚对象的生活补助，努力改善困难群众的生活。

　　六、重视人才培养，实施科教兴市战略

　　加快建设教育强市。深化教育管理体制和办学体制改革，鼓励和支持社会力量办学，积极发展民办教育。全面落实农村基础教育实行“以县为主”的管理体制，完善中小学“一费制”，优化教育结构，积极稳妥地调整中小学布局，建设示范性普通高中，发挥中等职业教育优势，培养高素质劳动者和实用型人才。整合我市高等教育资源，五邑大学要在提高办学质量、培养更多高素质人才的同时，加强与国内外高校、科研机构以及本地企业的合作，积极为地方建设服务。推进高等教育大众化，发展继续教育，构建终身教育体系。

　　进一步加强全社会的科技创新体系建设。建立以企业、高校及科研院所为主体的技术创新平台，加强创业载体建设，引导更多的大中型企业建立技术开发机构。促进科技型中小企业发展壮大。组织实施专业性的镇级技术创新示范工程。发挥科技中介机构的作用，发展技术市场。

　　加强人才队伍建设。大力引进熟悉ＷＴＯ规则的专才、懂行政管理和业务管理的复合型人才，以及工程技术人才、金融人才和法律人才。认真落实人才政策，建立人尽其才、能上能下、充满活力的用人机制，营造干事创业的良好环境，为加快发展提供强大的人才保障和智力支持。

　　七、发展社会各项事业，建设侨乡先进文化

　　加强社会主义精神文明建设。以全面提高人的素质为中心，组织开展创建省级文明城市活动。加强思想道德建设，弘扬“开拓创新、文明开放、勤劳创业、团结奉献”的侨乡精神，全面实施《公民道德建设实施纲要》，以诚实守信为重点，加强对广大干部群众特别是青少年的社会公德、职业道德和家庭美德教育，促进公民道德素质提高，树立昂扬向上的社会风尚。

　　积极发展文化事业和文化产业。围绕省委、省政府提出建设文化大省的目标，高水平建设好五邑文化广场、华侨博物馆、美术馆、电视中心、会展中心、体育公园、青少年宫、妇女儿童活动中心等文化娱乐基础设施。繁荣各项文艺创作，提升城市文化品位。进一步活跃广场文化，推动社区文化、企业文化、校园文化等群众文化活动的开展。坚持不懈地开展扫黄打非活动，营造文明开放、健康向上、奋发创业的侨乡文化氛围。办好２００３年江门艺术节。深化文化管理体制改革，整合侨乡文化资源，做大做强广播影视、新闻出版等重点产业，促进文化与经济的融合。

　　加强医疗卫生预防保健工作。以农村卫生、疾病预防控制、妇幼老年保健、社区卫生和公共卫生为重点，努力提高人民健康水平。积极推进医疗卫生改革，发展民营医疗机构，扩大药品集中招标采购，规范医疗收费。强化医疗卫生质量管理，坚决打击和取缔无证行医，规范医疗市场秩序。继续开展无偿献血活动。建立和完善新型农村合作医疗制度，加强农村卫生建设，推进改水、改厕和垃圾无害化处理工作。开展创建国家卫生城市、卫生城镇、卫生村活动，把全民爱国卫生运动提高到一个新水平。巩固防治非典型性肺炎的成果，建立疾病预防控制体系和突发公共卫生事件应急机制，提高我市疾病特别是传染病的控制防治能力。

　　加强人口与计划生育工作。稳定低生育水平，进一步提高人口素质。落实人口与计划生育目标管理责任制，做好计划生育基础工作，加强对流动人口计划生育的管理和服务，确保实现今年人口增长控制目标。

　　发展体育事业。实施《全民健身计划纲要》和《奥运争光计划纲要》，广泛开展全民健身活动，努力培养优秀体育人才，不断提高竞技体育水平。加强体育设施建设。办好２００３年世界台球锦标赛和江门市第四届老年人运动会。

　　与此同时，加强社会救济、社会互助、社会福利和优抚安置工作。抓好国防教育，做好征兵和复退军人安置工作，提高军民共建“双拥模范城”活动的水平。积极做好外事、侨务、台湾事务、人防、残联、宗教、老龄、档案、地方志等工作，促进各项社会事业的全面发展。

　　八、加强政治文明建设，提高民主法制建设水平

　　全面建设社会主义政治文明。深入推进依法治市工作，抓好普法教育，提高广大干部群众的民主意识和法制观念。加强行政执法队伍建设和行政执法监督，健全执法过错责任追究制度和行政管理公示制，提高行政决策透明度。各级政府要自觉接受人大的监督，切实办好人大议案、人大代表建议和政协委员提案，重大情况向人大报告，经常听取人民代表意见。主动听取工青妇、民主党派、无党派人士、工商联和人民团体的意见。加强人民来信来访工作。

　　加强社会治安综合治理。以建立防范机制为重点，严厉打击“黄、赌、毒”等违法犯罪活动，创建安全小区和“无毒社区”。持续开展以打击偷抢为重点的刑事犯罪专项斗争，坚决打击各种经济犯罪。加强政法队伍建设，提高司法人员和公安干警的整体素质。切实搞好安全生产和交通安全。严厉打击″法轮功″违法犯罪活动。及时疏导各种人民内部矛盾，维护社会稳定。

　　加强政府自身建设。落实机关作风建设领导责任制，建立健全监督机制。继续抓好领导干部廉洁自律、查处大案要案、纠正部门和行业不正之风三项重点工作，完善土地使用权出让、工程项目建设、政府采购和产权交易招投标制度，从源头上预防和治理腐败。进一步转变政府职能，增强政府工作透明度，自觉接受人民群众和新闻舆论的监督。推行电子政务，降低行政成本，提高行政效率。加强公务员队伍建设，强化勤政为民意识，建设廉洁高效、勤政务实的政府机关。

　　各位代表，未来的五年，是我市现代化建设在新的起点上向更高水平迈进的五年，任务光荣而艰巨。下一届政府将与全市人民一道，在市委的正确领导下，解放思想，抢抓机遇，开拓创新，团结奋进，努力开创工作新局面，为全面建设富民强市新侨乡而奋斗！
